# Voices from the past: results of the ESP history of pathology working group survey on pathology museums

**DOI:** 10.1007/s00428-022-03284-w

**Published:** 2022-01-26

**Authors:** Raffaella Santi, Roberta Ballestriero, Vincenzo Canzonieri, Jacek Gulczynski, Rosa Henriques de Gouveia, Aurelio Ariza, Lina Carvalho, Gabriella Nesi

**Affiliations:** 1 Hematopathology Unit, Careggi University Hospital, Florence, Italy; 2 Academy of Fine Arts of Venice, Venice, Italy; 3 Art Historian in Residence, Gordon Museum of Pathology, King’s College, London, UK; 4 Pathology Unit, Centro di Riferimento Oncologico di Aviano (CRO Aviano), IRCCS, National Cancer Institute, Aviano, Italy; 5 Department of Medical, Surgical and Health Sciences, University of Trieste, Trieste, Italy; 6 Department of Pathology and Neuropathology, Medical University of Gdansk, Gdansk, Poland; 7 Histology and Pathology, Faculty of Life Sciences, University of Madeira (UMa) & Clinical and Anatomical Pathology Laboratory (LANA), Funchal, Madeira Portugal; 8 Anatomical and Molecular Pathology Institute (IAP-PM), Faculdade de Medicina da Universidade de Coimbra (FMUC), Coimbra, Portugal; 9 Department of Anatomic Pathology, Hospital Germans Trias I Pujol, Autonomous University of Barcelona, Barcelona, Spain; 10 Division of Pathological Anatomy, Pathology Section, Department of Health Sciences, University of Florence, V.le G. Pieraccini 6, 50139 Florence, Italy

**Keywords:** Pathology museums, Anatomical collections, Biological archives, Medical education

## Abstract

While keeping their original purpose of training medical students, pathology museums hold great biological value, offering unique specimens for scientific research through modern radiological, pathological and biomolecular techniques. Moreover, the artefacts, models and drawings displayed in these museums are a precious cultural and artistic heritage. Preservation of the anatomical samples and maintenance of the facilities are neither easy nor inexpensive and call for patronage. The development of a European Pathology Museum Network would undoubtedly facilitate study, access and divulgation of antique pathology collections. Data from a survey conducted by the European Society of Pathology (ESP) History of Pathology Working Group have allowed creation of a comprehensive, multifaceted portrait of European university museums, reflecting their history, diversity, geography, institutional status, stakeholders, projects, professionals, audiences, policies and best practices.

## Introduction

Since its foundation in 2010, on the initiative of Professor Jan G. van den Tweel (1942–2020), the European Society of Pathology (ESP) History of Pathology Working Group has grown steadily, thanks to the commitment of all its members of different ages and from different scientific fields. The main goal of the Working Group is to spread the values of historical discoveries, messages and material, which the ESP acknowledges can inspire new generations of undergraduate and postgraduate students.

**Fig. 1:**
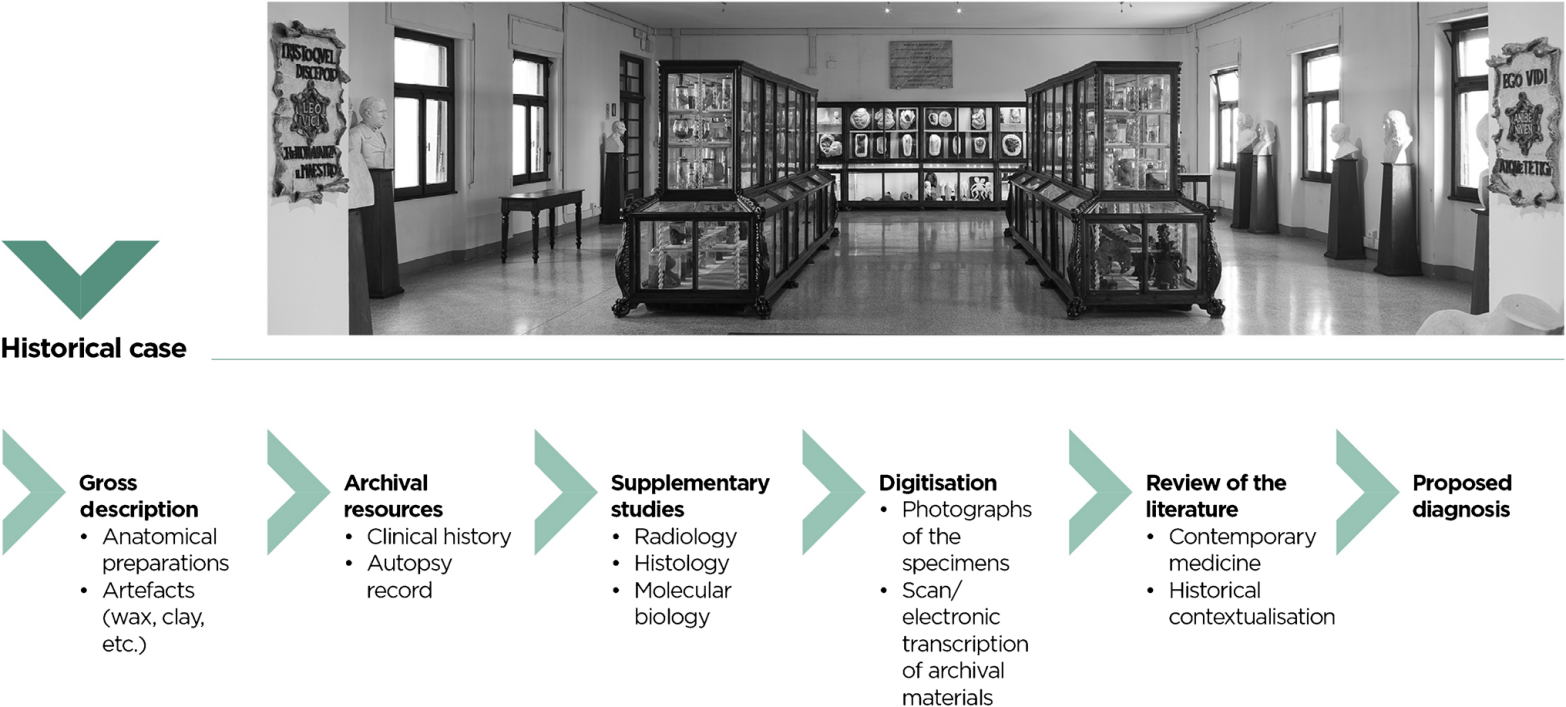
Representative scheme of the diagnostic approach to antique specimens/artefacts housed in pathology museums. In the foreground, overview of the Pathology Museum of the University of Florence, Florence, Italy (photograph by Lorenzo Mennonna)

Anatomy and pathology museums are no exception to the gradual loss of interest in scientific museology, and, without forgetting their conservative priorities, it is mandatory to keep in step with the times if they are to maintain their original didactic role [1]. During the first half of the twentieth century, pathology museums were a principal educational resource in medical schools. Since the dawn of modern medicine in the second half of the twentieth century, an ever-increasing body of knowledge has been progressively included in the medical curriculum and the teaching of gross pathology disparaged. Museums that have survived this cultural upheaval can be found all over the world, mainly in Europe. Several university-related collections are also located in North America, South America and Australia, such as the Maude Abbott Medical Museum in Montreal, the Johns Hopkins Medical Institutions (including the Department of Art as Applied to Medicine) in Baltimore, the Oswaldo Cruz Institute in Rio de Janeiro and the Harry Brookes Allen Museum of Anatomy and Pathology in Melbourne [2]. Many of these early “pathology classrooms” are still in use, reshaping and reinventing themselves to stay relevant in a digital world. The same technical ways and means of communication that now rival traditional museums can contribute to their innovation and improve access to ancient collections, through digital photographs and documentation recorded on appropriate appliances (CD-ROM, DVD) or shared via the World Wide Web [3]. For any anatomical specimen in the museum, a “route” may be contemplated covering patient clinical details, radiological images and histological findings, along with up-to-date literature on the topic. Scientific interpretation and contextualisation of the historical case will offer students a novel educational experience and scholars a chance for deeper investigation. To all intents and purposes, anatomical collections are “biological archives” open to research through state-of-the art imaging and biomolecular technologies (Fig. 1).

Preservation of specimens and facilities is neither easy nor costless and calls for patronage. The development of a *European Pathology Museum Network* would promote the study, access and divulgation of historical collections. The ESP History of Pathology Working Group has endeavoured to produce a comprehensive picture of the many facets (i.e. history, diversity, location, institutional status, existing networks and stakeholders, projects, professionals, audiences, policies, best practices and publications) of European pathology museums.

## General features of the survey

**Fig. 2:**
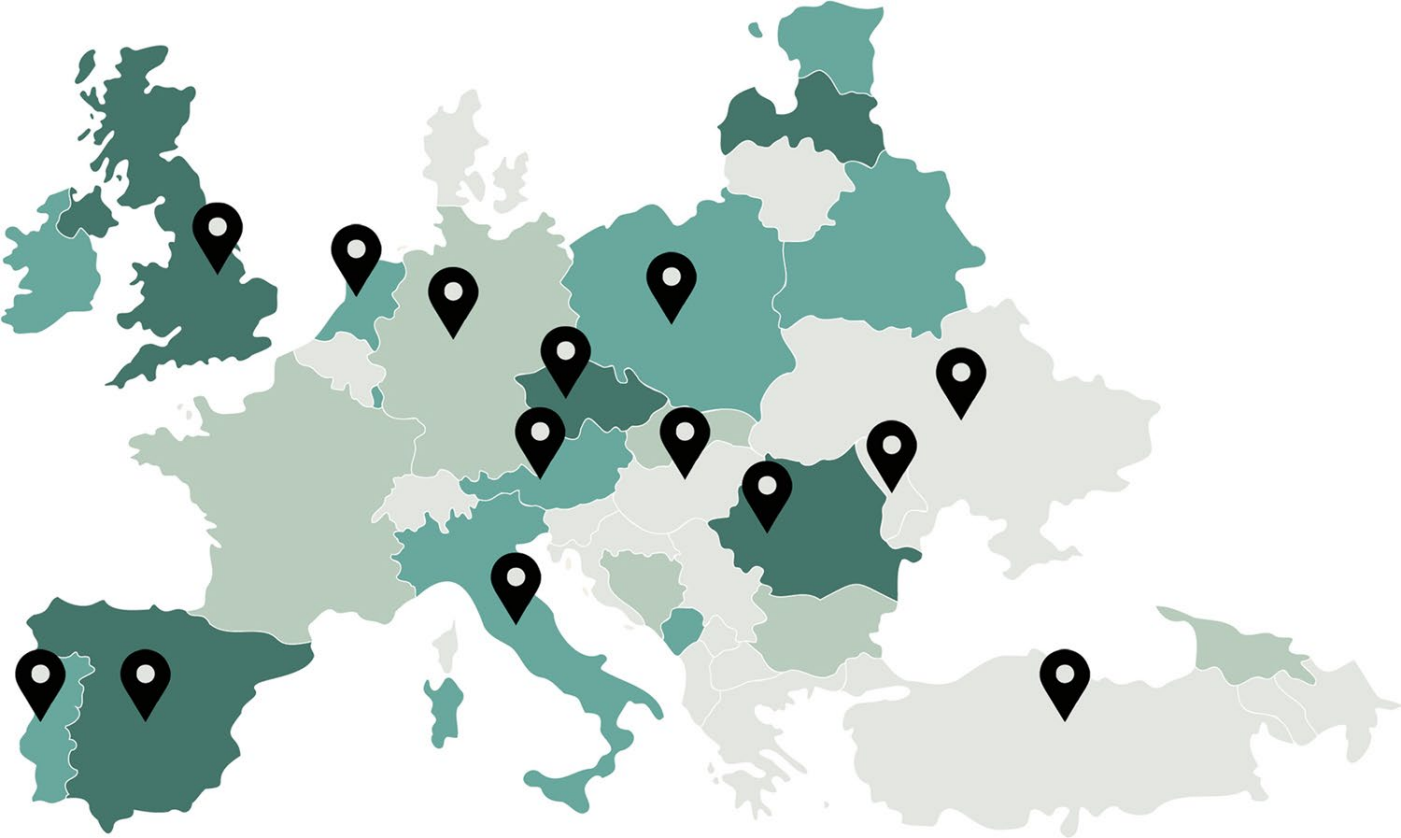
Map of the countries participating in the survey. Each pointer represents one or more participants

Twenty-seven members of the ESP History of Pathology Working Group, from 14 countries, participated in the study (Fig. 2). The online survey was available on the ESP website 2 years from May 2017. It comprised 24 closed- and open-ended questions (Table 1) concerning four main issues: (i) museum organisation and structure, (ii) type and number of objects, (iii) collection experiencing and sharing and (iv) strategies and challenges for contemporary pathology museums.

**Table 1:** Questionnaire used to conduct the ESP survey on pathology museums/collections (light grey boxes indicate open-ended questions)

**Q1** Name of museum
**Q2** If the collections are not your responsibility, please advise us who to contact, or forward the email invitation or questionnaire to the appropriate person
**Q3** Contact person:
**Q4** Address of museum/collection:
**Q5** Are you willing to be contacted to provide further information about this survey?
- Yes
- No
**Q6** Type of Museum (determined by its most important funding source):
- Independent
- University
- Other (please specify)
**Q7** How many collection staff (curators, conservators, collection managers, store people, etc.) are employed?
**Q8** Will this information be about the whole museum/collection?
- Yes
- No
- If so, please specify
**Q9** How many objects are there in the collections? Exclude inaccessible collections such as archives or bulk finds
- 1–500
- 501–1000
- 1001–5000
- 5001–10,000
- More (please specify)
**Q10** Genre of objects:
- Wet specimens
- Dry specimens
- Artificial models in wax, clay, glass, plastic, etc. (please specify)
**Q11** Are the collection specimens documented by original clinical records?
- Yes
- No
**Q12** What are the most important topics/issues your museum addresses?
**Q13** Is there a member of staff or an independent scholar conducting research on the collections?
- Yes
- No
- If so, please specify
**Q14** Can the public access the stored collections?
- Yes
- No
**Q15** If so, please tick all that apply:
- Visible storage as part of normal displays
- Advertised visits for public or for groups
- Pre-booked visits for groups
- By appointment -visitors can work with objects in store
- By appointment -objects are brought to visitors
- In a specially designed workplace for visitors
**Q16** Do you record the number of visitors accessing your collections each year?
- Yes
- No
**Q17** How do people access details of the contents of the collection? Please tick the three most widely used routes. If there are more than three, please add to “other”
- Online catalogue
- Internally published hard copy catalogue
- Externally published catalogue
- Telephone enquiry
- E-mail enquiry
- Written enquiry
- Word of mouth
**Q18** Are the collections listed online?
- Yes
- No
**Q19** Do you have plans to implement collection information online?
- Yes
- No
**Q20** Are there any plans to increase use of collections?
- Yes
- No
**Q21** If so, please tick relevant options below:
- Open storage
- Collections centre
- Regular tours
- More displays
**Q22** Which of the following are the main obstacles that hinder use of the stored collections? Please tick the three most important. If there are more than three, please add to “other”:
- Limited staff
- Limited resources
- Physical constraints of space
- Lack of money
- Poor collections documentation
- Security issues
- Preservation/conservation issues
- Lack of public interest
- Low strategy priority
- Other (give details)
**Q23** Would you like to comment on any part of the questionnaire?
- Yes
- If so, please specify
- No
**Q24** Is there anything else you think we should take into account?
- Yes
- If so, please specify
- No

## Museum organisation and structure

The vast majority of respondents are affiliated to academic institutions with the exception of one independent collection centre. Approximately 30% of pathology museums do not have dedicated employees. If they do, staff consists of one or two persons in 50% of cases and three or more in 23%.

For hundreds of years, educational and training programmes developed in pathology museums have been milestones along the path to becoming a physician. It was during the sixteenth and seventeenth centuries that specific spaces, housing anatomical and pathological specimens were instituted with the aim of teaching medical students [4]. Pathological collections substantially improved when the fixation techniques, devised by Johann Carl Kaiserling (1869–1942) and Leonhard Jores (1866–1935), allowed the natural colours of moist preparations to be maintained or restored for long-term display [5].

“He who attempts to teach anatomy without a museum”, stated the Scottish anatomist Frederick Knox (1794–1873), “strictly deserves the name of impostor” [3]. Museums contributed to promoting morbid anatomy as a distinctive specialty. By the middle of the eighteenth century, all eminent medical teaching institutions had a museum, and the curators evolved from “inspectors of the dead” to lecturers in morbid anatomy, or in some instances reached the more privileged title of “Professors of Pathology” [6]. The relationship between medical museums and pathology as a discipline is also highlighted in the seal of the International Academy of Pathology (IAP). The lettering “IAMM 1906”, inscribed below the microscope, globe and lamp of wisdom, stands for the International Association of Medical Museums, founded by Maude Abbott from McGill University in 1906, which eventually gave rise to the IAP in 1955.

With the use of the microscope for scientific purposes in the first decades of the nineteenth century, histopathology emerged as a new medical field and became the prerogative of pathologists [7]. Pathology was taught to medical students, who gained knowledge of gross pathology through the examination of museum specimens and of histopathology through the study of tissues under the microscope. Nowadays, hands-on practice of gross pathology and histopathology is seldom part of the curriculum for undergraduate students in medicine, and museums have become remote islands of mainly historical interest [8]. Conversely, the importance of pathology museums should be explained to medical students during pathology lectures and visits be encouraged. Indeed, with the decline of autopsy rates, direct observation of basic tissue processes (e.g. peptic ulcer disease, myocardial infarction) using gross specimens and/or artefacts held in the museum would constitute an incomparable learning experience. Comprehension of the dynamic course of diseases would also benefit from a morphology-based approach. Pathology museums, moreover, are key places to explore major developments in scientific thought and the impact of science on human culture and behaviours.

## Type and number of objects

**Fig. 3:**
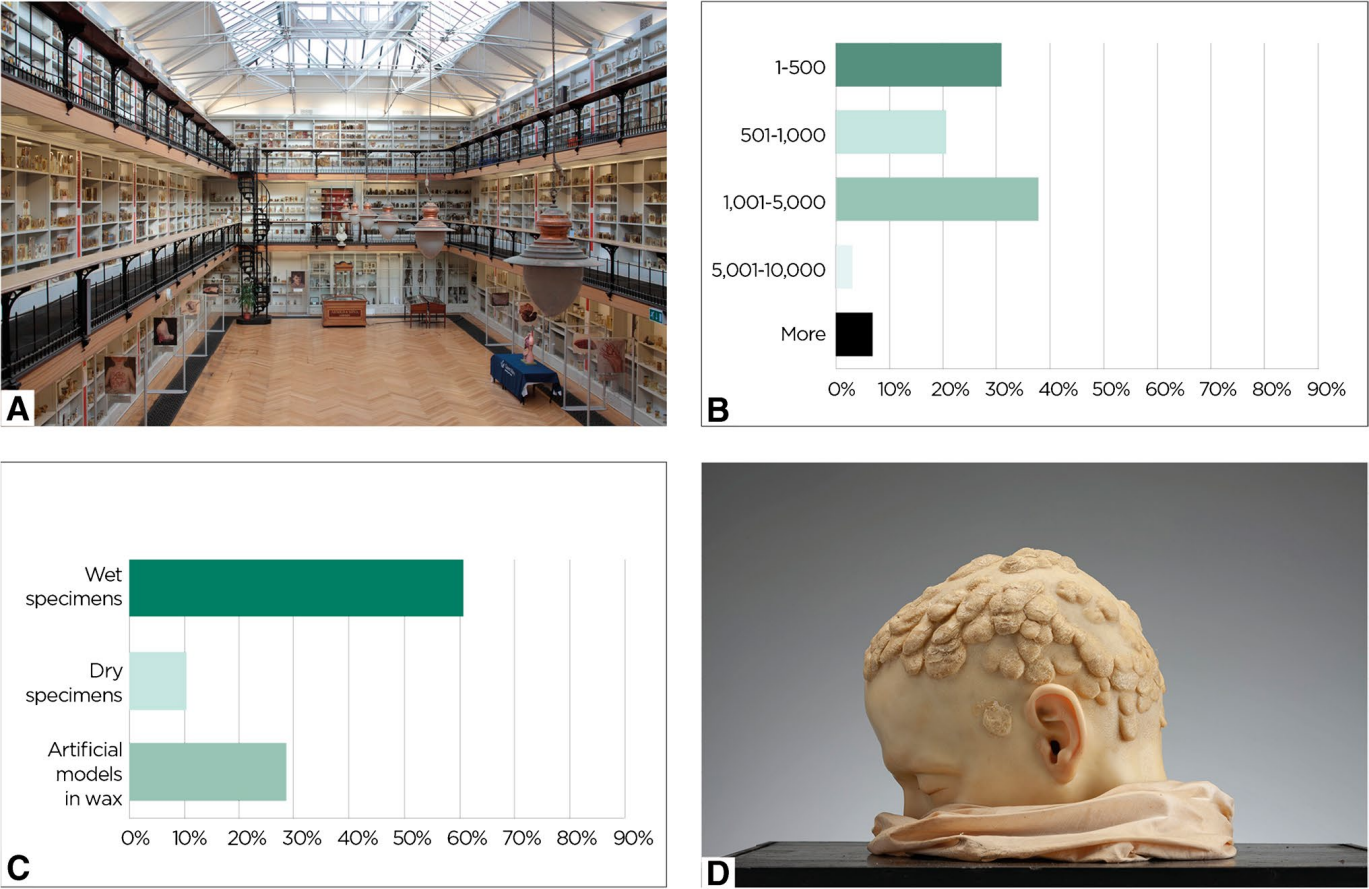
**A** The Gordon Museum of Pathology, King’s College London, United Kingdom. **B** Bar graph of survey results to the question: “How many objects are there in the collections? Exclude inaccessible collections such as archives or bulk finds”. **C** Genre of objects according to survey results (bar graph). **D** G*uttate psoriasis*, wax moulage by unknown wax artist, nineteenth century, Pathology Museum of the University of Florence (photograph by Lorenzo Mennonna)

Museum collections encompass wet/dry specimens and artificial models in various proportions, with the former being more frequent (Fig. 3). The majority of specimens (59%) are described using original clinical documentation. Approximately half of the institutions (52%) record up to 1000 objects, 38% from 1001 to 5000, and 10% as many as 10,000 or more. Various aspects of pathology are represented, ranging from congenital to neoplastic diseases; however some collections specialise in one or a limited number of medical branches, e.g. dentistry, dermatology and cardiology.

Anatomical and pathological specimens are usually preserved in formalin-based solution in fully labelled glass jars. Before adequate fixation techniques were available, the difficulties in conserving pathological material prompted the decision to set up duplicates in order to instruct young doctors without resorting to corpse dissection. Among the various materials by which models are made, wax takes pride of place. Wax models portraying normal and pathological anatomy are often hidden gems in scientific museums. The development of pathology as a scientific discipline was an important prerequisite to the art of moulaging [9, 10]. During the nineteenth century, wax models gave medical students practical knowledge of both normal and aberrant processes, not always possible with fixed organs and tissues. In addition to tridimensionality, moulages offered the “dimension” of colour, a key element for correct gross diagnosis. All the same, one should not underestimate the artistic value of these works.

Clinical histories and autopsy records are invaluable resources for scientific investigation into museum collections. Contextualisation of the medical knowledge of the time, enriched with fragments from the personal stories of patients, unveils the cultural value of these specimens. A detailed catalogue helps achieve the educational purpose of a collection, providing evidence of which organ systems and diseases are on display [11]. Without a catalogue, a museum loses its voice and therefore the possibility to recount the story behind an object.

## Collection experiencing and sharing

Public access is allowed in 48% of pathology museums. In approximately one third of these cases, stored collections are part of normal displays, with the remainder preferring to design museum experience as pre-booked visits (47%) and advertised events (13%), or having a specific workspace for the purpose (7%). The majority of institutions (54%) do not register the number of visitors. “Word of mouth” (53%) is the main means of communication regarding contents of the collections, while written/e-mail/telephone enquiries are the rule in 30%. A few museums rely on Internet resources (4%), and 13% hold a hard copy catalogue. Collections are listed online only in 12% of cases.

Multimedia and information technology has dominated medical education over recent years. Virtual microscopy courses and technically elaborated learning tools are in easy reach of pathologists and their students. Digitisation presents a great opportunity for scientific museums, providing access to materials via the web, minimizing damage to original items, increasing interest in these institutions and ensuring maximum public access to their collections [5, 12]. Indeed, one of the tasks of a pathology museum is to convey human pathology to the general public. It is one thing to say that “smoking can seriously harm your health”, but quite another to show the real lung of a smoker and the consequential damage caused to its structure.

## Strategies and challenges for contemporary pathology museums

Approximately 60% of institutions plan to implement collection information online, and a higher proportion of responders (86%) aim to increase use of collections. In particular, strategies to improve the use of collections include establishing a collection centre (38%), allowing open storage (29%), scheduling regular tours (15%) and organising more displays (18%). Unfortunately, the general feeling is that innovation is hampered by limited financial resources (25%), staff shortages (25%) and lack of space (18%).

Due to difficulty in sourcing new samples to museum exhibits, there has been no increase in many collections, whose integrity is threatened by the inherent fragility of anatomical preparations [13, 14]. Curators are therefore committed to preserving antique specimens while ensuring their availability for current research projects. Recently, the role of imaging techniques, such as nuclear magnetic resonance (NMR) and computed tomography (CT), has been highlighted in the study of osteological preparations and specimens in fixative liquid, which no longer need to be removed from their original glass jars [15–17]. A digital library of radiological images could offer an efficient answer to conservation problems. It could also serve as a useful tool in diagnostic definition of museum specimens and, more generally, in teaching anatomo-radiological correlations [17]. Furthermore, the application of modern immunohistochemical and molecular techniques to historical long-term preserved fixed tissues has proven feasible, opening new lines of research [18, 19].

Antique anatomical collections allow the study of disease aetiology and pathomorphosis in relation to the profound changes in socio-economic conditions of resident populations over the centuries. Most preparations date back to the pre-industrial or pre-antibiotic era, and it is now common knowledge that genome-environment interactions play a crucial role in human carcinogenesis. Molecular investigations on ancient tumours can help shed light on the history of cancer and the relationships between genetic alterations, lifestyle and environmental risk factors [20]. Pathology museums may also contribute to the study of occupational diseases that paralleled the Industrial Age, illustrating the effects of toxic substances such as lead and arsenic. These collections are therefore valuable not only for their original educational purposes but also for their documentation of diseases already eradicated or rendered infrequent by improved diagnosis and treatment.

Management, custody and display of human remains entail a vast amount of responsibility regarding several ethical and legal issues. Historical museum exhibitions may reflect the attitude of Western Countries prior to the recognition of human rights and respect for other cultures [21]. Far from bona fide research purposes, putting race as a defining category for human specimens had supported pseudoscientific theories of discrimination. Museum curators should offer appropriate tools to view collections in their historical and cultural context, endorsing inclusion and dialogue among cultures in a perspective of mutual understanding and enrichment.

## Conclusions

Pathology museums hold materials unique in displaying anatomy and disease in three dimensions. They generally encompass different types of collections, each integral to the other in outlining a cognitive path. This body of evidence, re-interpreted on the basis of current medical practice and implemented by radiological, histological and biomolecular data, allows a more accurate diagnostic definition of antique anatomical preparations and the assessment of new museological strategies.

Promotion of these institutions relies on standardised approaches of conservation and cataloguing, but introduction of multimedia and interactive systems is equally essential. Permanent digital archives of anatomical collections should be created, thereby facilitating research and cooperation networking. Cultural heritage is the legacy that we attain from the past, experience in the present, and convey to future generations.
