# Correction to: Voices from the past: results of the ESP history of pathology working group survey on pathology museums

**DOI:** 10.1007/s00428-023-03686-4

**Published:** 2023-10-31

**Authors:** Raffaella Santi, Roberta Ballestriero, Vincenzo Canzonieri, Jacek Gulczynski, Rosa Henriques de Gouveia, Aurelio Ariza, Lina Carvalho, Gabriella Nesi

**Affiliations:** 1 Hematopathology Unit, Careggi University Hospital, Florence, Italy; 2 Academy of Fine Arts of Venice, Venice, Italy; 3 Art Historian in Residence, Gordon Museum of Pathology, King’s College, London, UK; 4 Pathology Unit, Centro di Riferimento Oncologico di Aviano (CRO Aviano), IRCCS, National Cancer Institute, Aviano, Italy; 5 Department of Medical, Surgical and Health Sciences, University of Trieste, Trieste, Italy; 6 Department of Pathology and Neuropathology, Medical University of Gdansk, Gdansk, Poland; 7 Histology and Pathology, Faculty of Life Sciences, University of Madeira (UMa) & Clinical and Anatomical Pathology Laboratory (LANA), Funchal, Madeira Portugal; 8 Anatomical and Molecular Pathology Institute (IAP-PM), Faculdade de Medicina da Universidade de Coimbra (FMUC), Coimbra, Portugal; 9 Department of Anatomic Pathology, Hospital Germans Trias I Pujol, Autonomous University of Barcelona, Barcelona, Spain; 10 Division of Pathological Anatomy, Pathology Section, Department of Health Sciences, University of Florence, V.le G. Pieraccini 6, 50139 Florence, Italy

**Correction to: Virchows Archiv (2022) 480:1231-1238**

10.1007/s00428-022-03284-w

In this article, the author's surname, Jacek Gulczynski, was misspelled as ‘Gulcznsky’

The original article has been corrected.

